# The Effect of Ni-ZSM-5 Catalysts on Catalytic Pyrolysis and Hydro-Pyrolysis of Biomass

**DOI:** 10.3389/fchem.2020.00790

**Published:** 2020-09-25

**Authors:** Ya-Long Ding, Hua-Qin Wang, Mei Xiang, Pei Yu, Rong-Qiang Li, Qing-Ping Ke

**Affiliations:** ^1^College of Chemistry and Pharmaceutical Engineering, Huanghuai University, Zhumadian, China; ^2^School of Chemical Engineering and Materials, Changzhou Institute of Technology, Changzhou, China; ^3^College of Chemistry and Chemical Engineering, Anhui University of Technology, Ma'anshan, China

**Keywords:** lignocellulose, microporous, Ni-ZSM-5, catalytic pyrolysis, hydro-pyrolysis

## Abstract

With the demand of energy and re-utilization of wastes, the renewable lignocellulosic biomass, has attracted increasing and significant attention for alleviating the growing energy crisis and environment problems. As main components of lignocellulosic biomass, lignin, cellulose, and hemicellulose are connected by hydrogen bond to form a compact skeleton structure, resulting the trenchant condition of biomass pyrolysis. Also, pyrolysis products of above three main constituents contain a large amount of oxygenates that cause low heating value, high corrosiveness, high viscosity, and instability. Meanwhile, zeolites are of considerable significance to the conversion of lignocellulosic biomass to desirable chemical products on account of fine shape selectivity and moderate acid sites and strength. Among numerous zeolites, ZSM-5-based catalysts have been most extensively studied, and the acidity and porosity of ZSM-5 can be tuned by changing the content of Si or Al in zeolite. Beyond that, doping of other metal elements, such as Mn, Co, Ni, Ga, Ce, Pt, into ZSM-5 is also an efficient way to regulate the strength and density of acid sites in zeolite precisely. This review focused on the recent investigation of Ni-modified microporous ZSM-5 used in catalytic pyrolysis of lignin and cellulose. The application of metal-modified hierarchical ZSM-5 is also covered.

## Introduction

With the rapid development of world economy and the continuous increase of population, human is facing unprecedented energy demand (Department of Economic Social Affairs United Nations, 2019; Energy Information Administration United States, 2019). Petroleum-based resources are currently society's primary source of chemicals and transportation fuels. However, due to limited oil reserves, uneven geographical distribution and environmental motivation to reduce carbon dioxide emissions and other factors, renewable energy such as lignocellulosic biomass has become a promising candidate resource for the production of renewable fuels and chemicals (Huber et al., 2006; Demirbas, 2008; Saxena et al., 2009; Zakzeski et al., 2010). Although fossil fuels continue to meet most of the world's energy demand, renewable energy is the world's fastest growing form of energy and its worldwide consumption will increase by 3% per year between 2018 and 2050 (Energy Information Administration United States, 2019). As carbon-neutral and the most abundant renewable energy resource for production of biofuels and valuable chemicals, biomass energy has attracted great attention, and developed rapidly in the last two decades (Jürgen, 2006; Ragauskas et al., 2006; Chheda et al., 2007; Huber and Corma, 2007). Biomass consists of a mixture of strongly bonded natural polymers such as cellulose (40–50%), hemicelluloses (25–30%), lignin and non-carbohydrate (Zhang et al., 2006; Menon and Rao, 2012). One most recent emerging focus of biomass utilization was the use of bio-oil generated from sustainable biomass feedstocks.

Biomass conversion is generally carried out in two ways: thermochemical decomposition, including gasification, biological carbonization, liquefaction, thermal decomposition (pyrolysis) process, and biological digestion process (essentially microbial digestion and fermentation) (Rocca et al., 1999; Lewandowski et al., 2011). Biomass conversion through thermochemical processes is considered to be a very promising biofuel carrier, because obtained bio-oil can be easily transported, burned directly in a thermal power station or gas turbine, and used in traditional refineries to produce high-quality light hydrocarbon fuels. Fast pyrolysis technology, firstly appeared in the late 1970s, is a promising way to convert biomass into bio-oil, for its high liquid oil production (up to 80% of dry feed) (Bridgwater and Peacocke, 2000; Onay, 2007). Bio-oil has many potential applications in the field of energy including being used for static application, and also can be upgraded to transportation fuel (Bridgwater and Cottam, 1992; Czernik and Bridgwater, 2004; Bridgwater, 2012). However, bio-oil usually presents several shortcomings, among which high water and oxygen content, corrosiveness, heat storage instability, immiscibility with petroleum fuel, high acidity, high viscosity, and low calorific value are main obstacles for its direct application as fuel (Michael, 2008; Park et al., 2011). The major product from non-catalytic fast pyrolysis are acids including linoleic acid, nitrogen containing compounds such as amines, amides, indoles, and pyrroles. Other organic compounds are ketones, alcohols, esters, ethers, phenols, and sugars. Acid is related to the high acidity of bio oil, and ketone is the cause of the instability of bio oil. Ethers and esters will reduce the heating values of bio oil, and nitrogen compounds cause environmental problems. In order to improve fuel quality, the process used must remove oxygen, convert carboxylic acid and other active substances into milder products, and add hydrogen to bio oil (Corma et al., 2007a; Gallezot, 2007; Alonso et al., 2010). Catalytic pyrolysis is the process of pyrolysis with assistance of catalyst and recombination of organic steam obtained from rapid pyrolysis of biomass into a class of compounds under the action of catalysts. Meanwhile, liquid fuels such as aromatic compounds within the gasoline range can be directly pyrolyzed by biomass materials (Chen and Degnan, 1988). However, due to the existence of lignin and hemicellulose structure, it is difficult to transform lignocellulose into liquid fuels by simple chemical or enzymatic hydrolysis, and finally into biofuels. Many pyrolysis parameters, such as temperature, pressure, heating rate, reactor structure, biomass type, particle size, and so on, have been widely studied and summarized (Melligan et al., 2011; Bridgwater, 2012).

Catalysts play an important role in the upgrading of bio-oil (Horne and Williams, 1996; Zhang et al., 2011). The primary catalysts mainly consist of supported noble metals Pt and Pd, transition metals Mo, Ni-Mo, Co-Mo, Ni-W, and Co-W (Viljava et al., 2000; Olivas et al., 2008; Yang et al., 2008). Although the noble metal catalysts have excellent catalytic performance, their high cost limit their large-scale application (Kubičková et al., 2005). To maintain the activity and stability of catalysts modified with metal Co and Ni, sulfurization of these catalysts is necessary during catalysts activation and bio-oil upgrading process, which will result in sulfur pollution of final products (Ryymin et al., 2010). In order to prevent sulfur pollution, it is of great significance to explore the non-sulfurized transition metal catalyst (Hu et al., 2018). ZSM-5 has been widely applied in crude oil refinery and gas adsorption-separation industry due to its strong acidity as well as shape selectivity. ZSM-5 is a kind of crystalline aluminosilicate material, which has unique two-dimensional channel-like pore structure with intersecting channels of ~0.55 nm in diameter favoring hydrocarbons of <10 carbon atoms, good thermal and hydrothermal stability, strong acid resistance, and carbon deposition resistance, adjustable acidity (Brönsted acid sites and Lewis acid sites), excellent shape selectivity, isomerization, hydrodeoxidization, and other catalytic properties (Sharma and Bakhshi, 1991; Chen et al., 2020). It has been successfully used in the hydrolysis of lignocellulose (Corma et al., 2007b; Mortensen et al., 2011; Park et al., 2011). ZSM-5 has also been widely used as a catalyst for biomass pyrolysis, and found to greatly change the composition of bio oil via dramatically reducing oxygenated compounds by deoxidization reactions, increasing aromatic species, and producing more organic matters (bio-oil) which can be upgraded to gasoline and diesel fuel (Vitolo et al., 2001; Cheng and Huber, 2011; Mortensen et al., 2011; Park et al., 2011; Taarning et al., 2011). Oxygen is removed through formation of water due to strong dehydration tendency promoted by the strong acidity of H^+^-exchanged ZSM-5 (HZSM-5) (Topsøe et al., 1981; Kapustin et al., 1988; Triantafillidis et al., 2001; Lappas et al., 2009). However, the strong acidity of ZSM-5 may lead to the decrease of organic components of bio oil by over-cracking to hydrocarbon, gas, or coke. Therefore, tuning of the acid sites is essential to utilization of catalyst (Huang et al., 2009).

Transition metal dopants are believed to affect the oxygen exclusion pattern by producing more carbon oxides and less water, so that more hydrogen can be incorporated into hydrocarbons. Different metal-modified ZSM-5 catalysts (Ce-ZSM-5, Co-ZSM-5, FeZSM-5, Ga-ZSM-5, HZSM-5, Ni-ZSM-5) have been used in biomass pyrolysis to verify whether these metal promoted low acid zeolites produce higher hydrocarbon yield and less coke than the commercial ZSM-5 catalysts previously tested (Park et al., 2007; French and Czernik, 2010; Valle et al., 2010; Cheng et al., 2012; Neumann and Hicks, 2012). Among them, French and Czernik (2010) studied 40 different catalysts and found that Ni-ZSM-5 catalyst has the highest hydrocarbon yield. The incorporation of transition metals (such as nickel) can increase the yield of aromatics, and they found that the hydrothermal stability of ZSM-5 will be improved by metal impregnation (Valle et al., 2005). Considering the importance of hydrodeoxygenation of bio-oil for biomass utilization and the hydrogenation ability of transition metals such as Ni, this paper mainly reviews the research progress of Ni supported ZSM-5 zeolite in catalytic conversion of biomass, and comprehensively summarizes the following aspects: (1) the catalytic conversion of biomass promoted by single metal Ni supported ZSM-5 in the absence of hydrogen; (2) the promotion of Ni supported ZSM-5 in biomass catalytic conversion in hydrogen atmosphere; (3) the utilization of bimetal supported ZSM-5 composed of Ni and other metals in biomass catalytic conversion.

## Ni Modified ZSM-5

The most representative organic compounds in pyrolytic bio-oil can be divided into 13 major functional groups: aromatic hydrocarbons, aliphatic hydrocarbons, phenols, furans, acids, esters, alcohols, ethers, aldehydes, ketones, polyaromatic hydrocarbons (PAHs), nitrogen compounds, and heavier compounds (Iliopoulou et al., 2012). Among them, aromatic hydrocarbon, aliphatic hydrocarbon, and alcohol are considered as ideal biofuel products, and phenols and furans are considered as high value-added chemicals. Carbonyl compounds, such as acids, ketones, aldehydes, ketones, esters, ethers are undesirable products for their relation to corrosiveness, instability and reduce the heating value of bio oils. Other undesirable products includes polyaromatic hydrocarbons and nitrogen compounds due to environmental reasons.

The aromatics produced by catalytic pyrolysis can be divided into two types including (1) monocyclic aromatics (MAHs), such as benzene, toluene, ethylbenzene (EB), xylene, indene and other substituted benzenes as styrene, ethyl-methyl-benzene, etc. (2) polycyclic aromatics (PAHs: naphthalene, anthracene, phenanthrene, fluorene). The metal on ZSM-5 helps to catalyze the formation of monocyclic aromatics and retards the further polymerization of PAHs and other oxygenates, which is a competitive reaction and leads to the formation of PAHs.

The conversion of biomass to bio oil (X_A_) and selectivity to product *i* (S_i_) was defined as:

$$\begin{array}{l} {\;X}_{A}\left(wt.\%\right)=\frac{w_{0}-w_{1}}{w_{0}}\times 100\%\\ S_{i}\left(mol.\%\right)=\frac{n_{i}\times a_{i}}{\sum_{1}^{i}n_{i}\times a_{i}}\times 100\% \end{array}$$

Where *w*_0_ refers to the initial weight of biomass, *w*_1_ means the weight of bio oil after catalytic pyrolysis; *n*_*i*_ refers to moles of the product *i*, and *a*_*i*_ is the carbon atom numbers for product *i*.

The addition of Ni metal to ZSM-5 zeolite is believed to promote the conversion of oxygenated and nitrogen compounds to aliphatics and aromatics and improve the hydrothermal stability of the catalyst, due to the synergistic effect of the dehydrogenation activity of nickel and the moderate acid strength of the catalyst (Valle et al., 2010). The formation of aliphatic compounds may be due to the fact that the metal part slows down the reaction between aromatics and other oxygenated compounds during the catalytic pyrolysis, forming alkylated benzene or polycyclic aromatic hydrocarbons (PAHs) (Carlson et al., 2010; Thangalazhy-Gopakumar et al., 2012).

### Monometallic Ni-Modified ZSM-5

Huynh et al. (2014) synthesized Ni-ZSM-5 for 4, 12, 21 wt.% Ni loadings. The N_2_ adsorption studies revealed a steady decline in adsorption capacities with the increase in Ni content, as the N_2_ adsorbed volume were 94.5, 75.2, 73.4, and 65.4 cm^3^·g^−1^ at standard temperature and pressure for 4, 12, 21 wt.% Ni loadings ZSM-5, respectively. This finding could be attributed to pore blocking by the increasing proportion of loaded metal species, which is in consistence with the results of Gayubo et al. (2010). The H_2_ temperature-programmed reduction (TPR) profile of 21 wt.% Ni-ZSM-5 consists of two peaks at low temperature (350–450°C) and high temperature (500–600°C). There are two possible explanations for this result. One is that nickel oxide can be reduced to two-step reaction as shown in formula (1) and formula (2), as Hadjiivanov et al. (2002) confirmed. The other explanation is the intensities of interaction between Ni^2+^ and HZSM-5 at different positions are different, which may lead to different reducibility, which is in line with conclusion from Maia et al. (2010). According to the elemental compositions analysis by X-ray photoelectron spectroscopy, the surface Ni/Si ratio decrease from 0.24 to 0.11 during the reduction of 21 wt.% Ni-ZSM-5, and this result may be due to the sintering or migration of Ni into the pore (volume) during reduction. Results from IR spectra of neat and reduced 21 wt.% Ni-ZSM-5 showed that the total acidity remains the same (619 and 588 μmol·g^−1^ for neat and reduced 21 wt.% Ni-ZSM-5). However, after impregnation with Ni species, the overall Brönsted acidity significantly decreased from 451 to 243 μmol·g^−1^ whereas the overall Lewis acidity increased from 168 to 345 μmol·g^−1^. The variation of overall Brönsted acidity and Lewis acidity may be attributed to substitution of protons at Brönsted sites by exchangeable cations, that is Ni^2+^ (Huynh et al., 2014). The overall Lewis acidity could be produced by dehydration of protonated oxide bridge after high temperature treatment in the calcination or reduction step (Hooff and Roelofsen, 1991). This combination of Brönsted acid sites and Lewis acid sites is more suitable for HDO reaction because the acid center can cleave C-O bond through protonation, while the metal center can activate H_2_ and reduce aromatic ring.

(1) $$\begin{array}{l} 2\;NiO+H_{2}\to Ni_{2}O+H_{2}O \end{array}$$

(2) $$\begin{array}{l} Ni_{2}O+H_{2}\to 2\;Ni+H_{2}O \end{array}$$

#### Catalytic Pyrolysis

Catalytic pyrolysis with cracking catalyst is an emerging technology to directly convert the oxygenated compounds produced in the pyrolysis of biomass into hydrocarbons, so as to improve the quality of bio-oil. Catalytic pyrolysis can be carried out in the atmosphere without high hydrogen pressure, which reduces the operation cost. The volatile oxygenated species in the pyrolysis gas enter into ZSM-5 pores and react with the protons in the active sites through dehydration, decarboxylation, decarbonylation, oligomerization, and dehydrogenation to generate aromatic compounds, carbon monoxide, carbon dioxide, and water.

The results from Huynh et al. revealed that phenol conversion of 14, 82, and 98% were obtained with 4, 12, 21 wt.% Ni-ZSM-5, respectively. For 21 wt.% Ni-ZSM-5, selectivity toward deoxygenated products reached 98%, which included ~88% for cyclohexane and 8% for benzene, indicating that the combination of metal and acid is needed to effectively remove oxygen from phenol. Tuning of the metal sites can provide additional control over the severity of hydrogenation (whether it is an attack on a substituent or aromatic ring system) and selectivity. Iliopoulou et al. (2012) investigated the catalytic upgrading of biomass pyrolysis vapors using different loadings (1, 5, 10 wt.%) Ni-modified commercial equilibrium ZSM-5 diluted with silica-alumina (containing 30 wt.% crystalline zeolite) via typical wet impregnation method. There is no significant loss of surface area at low loadings (1 and 5 wt.%). However, high loading (10 wt.%) results in a 15% decrease of surface area, which is mainly due to the blocking of micropores in ZSM-5 crystal when metal phases are formed (Vitolo et al., 2001). On the other hand, compared with porosity, the effect of metals on the acidity of ZSM-5 zeolite catalyst is more significant. The presence of 1 and 5 wt.% Ni reduced the number of Brönsted acid sites by 40%, and higher loading (10 wt.%) induced further reduction (47 wt.%). The significant decrease of Brönsted acid sites indicated that the acidic protons in ZSM-5 zeolite were exchanged by Ni ions during the dry impregnation process, and the quantity of Lewis was increased by 2–3 times, possibly due to the formation of the corresponding oxide, i.e., NiO, which can serve as Lewis acid center. Basing on X-ray diffraction (XRD) pattern, crystallite size of NiO increases from 28.5 to 39.5 nm for the 5 and 10 wt.% loaded catalyst, while it is difficult to identify the XRD peaks caused by NiO in Ni (1%) ZSM-5 catalyst. For10 wt.% Ni-ZSM-5, the rectangular or cubic NiO particles were highly dispersed on catalysts and showed a mean size of ~40–45 nm.

The results of deoxy-liquefaction of laminaria japonica to liquid oil over Ni-ZSM-5 showed that Ni-ZSM-5 catalyst could increase the liquid oil yield and the contents of aromatics and long-chain alkanes, and decrease the amounts of phenols, other oxygen and nitrogen containing species (Li et al., 2013). Vichaphund et al. (2015) investigate the catalytic pyrolysis of jatropha residues including 59.2% cellulose, 18.0% hemicelluloses, and 22.8% lignin with 3 wt.% Ni-ZSM-5 catalysts. The combination of Ni and acid sites provides an ideal environment for the oligomerization, cyclization, and dehydrogenation of small olefins, and improves the formation rate of aromatic compounds. The main product is toluene with selectivity of 36.4% while the selectivity of benzene is only 10%, owing to the alkylation of benzene and other oxygenated compounds to form alkylated benzene. In addition, the 3 wt.% Ni-ZSM-5 catalysts with different preparation method of ion-exchange and wet impregnation are compared in catalytic pyrolysis of jatropha residues. For ion-exchanged HZSM-5, the MAHs selectivity is 85–88% and PAHs can be reduced to 12–16%, while for impregnated HZSM-5, the MAHs selectivity is 85–90% and PAHs can be reduced to 10–15%.

The upgrading of bio-oil with Ni modified hierarchical ZSM-5 catalyst is also studied, and results showed that the preferential mechanism for O-removal using hierarchically structured Ni-ZSM-5 zeolite catalysts seems to proceed through decarbonylation and decarboxylation reactions at the Lewis acid sites evolved after metal incorporation (Veses et al., 2016). However, the main product in obtained bio-oil is phenols (42.8 wt%). Chen et al. synthesize hierarchical ZSM-5 catalyst by NaOH desilication and HCl treatment and investigate the catalytic pyrolysis of rice straw to aromatics with Ni modified ZSM-5. High yields of aromatics (28%) can be obtained at very low Ni loading (0.1 wt.%), and there is no difference in the selectivity of aromatic products when the amount of Ni added is 0.1, 0.5, or 1.0% (Chen et al., 2018). The study on effect of reaction temperature on conversion of phenol with hierarchical Ni-ZSM-5 shows that the main products were mostly benzene and cyclohexene formed by phenol deoxygenation at low and intermediate temperatures (393 and 423 K) whereas a shift in the reaction pathways occurred at higher temperature (448 K), leading to high slectivity of valuable alkylphenols (mostly cresols and cyclohexylphenols) generated by the occurrence of alkylation reactions catalyzed by the zeolite acid sites (García-Minguillan et al., 2020). The mixture of microporous ZSM-5 and another mesoporous zeolite is also employed for catalytic pyrolysis of biomass. Hu et al. (2020) investigate the catalytic co-pyrolysis of seaweeds and cellulose using mixed ZSM-5 and MCM-41, the yield of main product (furans) is 52.2%, which is higher than that using single ZSM-5 or MCM-41. Because of the interaction between the free radicals of seaweeds/cellulose and ZSM-5/MCM-41, as well as the synergism between the joint channel advantage of mesoporous molecular sieve and the acidity of microporous molecular sieve, the yield and composition of bio oil are improved.

The biomass pyrolysis experiments were performed on a bench-scale fixed bed tubular reactor, and catalytic pyrolysis experiment with NiO was also conducted for comparison (Iliopoulou et al., 2007; Triantafyllidis et al., 2007). Compared with the non-catalytic experiment, all the catalysts reduced the total liquid yield, increased the gas products and coke at the expense of organic yield. This behavior is due to various hydrocarbon conversion reactions, such as cracking, dehydrogenation, and cyclization/aromatization, which are catalyzed by zeolite protonic acid sites. Besides that, water formation was promoted by dehydration/decarboxylation of oxygenated compound on acid sites of ZSM-5 (Lappas et al., 2009). What is needed to point out is that the addition of Ni in to ZSM-5 does not influence the formation of water and leads mainly to increased production of H_2_ and C_2_-C_6_ gaseous hydrocarbons. Iliopoulou et al. (2012) suggested two important reaction routes: (1) bio oil deoxidized by decarboxylation, producing a small amount of water and *in situ* generating hydrogen; (2) *in situ* generated hydrogen atoms participate in the hydrogen transfer reaction on NiO atoms, increasing the generation of saturated hydrocarbon through the carbonium intermediate on the acid sites of zeolite. The almost unaffected water production indicates that the oxygen of bio oil compounds is not removed by hydrogen deoxidation, which can be promoted by Ni supported metals, but mainly by decarboxylation reactions. The increase of aromatics may be due to the enhancement of dehydrogenation of Ni metal, and the increase of phenolic content may be related to the decrease of Brönsted acid sites which are covering by Ni metal ions. After pyrolysis experiment, a majority of NiO particles converted to metallic Ni particles which showed smaller spherical or rectangular particles with an average size of ~17 nm, and the catalyst particle were covered by a layer of graphitized carbon, which may be due to the strong dehydrogenation effect of Ni species (related to the formation of coke) during pyrolysis. This is the first report of observation of reduction of Ni oxides to metallic Ni during biomass catalytic pyrolysis. And this result may play an important role in commercial use of Ni-ZSM-5, because the catalyst will continue to circulate between the pyrolysis reactor (reduction of Ni oxides) and the regenerator (oxidization of metal Ni) which is used to burn off char (coke deposited on the catalyst and possibly unreacted biomass). In addition, the *in-situ* formation of reduced Ni metal phase can also promote the hydrogen transfer reaction using the *in situ* produced H_2_ on metallic Ni or external supplied H_2_ in a hydro-pyrolysis process (Yung et al., 2016). Porosity and acidity of different Ni-ZSM-5 catalysts and their catalytic pyrolysis performance are shown in Table 1.

**Table 1:** Porosity and acidity characteristics of catalysts and catalytic pyrolysis performance of different catalysts.

**Catalyst**	**Surface area (m^2^/g)^a^**	**Acidic properties (μmol/g)**			**Raw material**	**Primary product in liquid phase (wt.%)**	**References**
		**Brönsted acidity**	**Lewis acidity**	**Total acidity**			
ZSM-5^b^	138	36.5^c^	18.1^c^	54.6^c^	Beech wood^d^	Phenols (35.0)	Iliopoulou et al., 2012
5 wt.% Ni-ZSM-5^b^	132	21.9^c^	54.6^c^	76.5^c^	Beech wood^d^	Phenols (39.0)	Iliopoulou et al., 2012
Ni-ZSM-5^e^	306	None	None	401^f^	Laminaria japonica	C_12_-C_21_ alkanes (22.9) Phenols (23.0)	Li et al., 2013
3 wt.% Ni-ZSM-5^g^	608	None	None	None	Jatropha residues	Aromatics (30.6), Acid (15.7) N-compounds (28.9)	Vichaphund et al., 2015
3 wt.% Ni-ZSM-5^h^	358	None	None	None	Eucalyptus urophylla	BETX^i^ (NM^j^) Naphthalenes (NM^j^)	Schultz et al., 2016
12 wt.% Ni-ZSM-5	386	None	None	None	Prairie cordgrass	C_4_-C_12_ hydrocarbons (32.45)	Cheng et al., 2016
1 wt.% Ni-ZSM-5	500	148^c^	270^c^	418^c^	Pine wood	Phenols (42.8)	Veses et al., 2016
0.1 wt.% Ni-ZSM-5^k^	None	None	None	None	Rice straw	Aromatics (28)	Chen et al., 2018

a *Multi-point BET method*.

b *Commercial equilibrium ZSM-5 zeolite catalyst diluted with silica-alumina (~30 wt.% zeolite)*.

c *Fourier-Transform Infrared (FTIR) measurements*.

d *A commercial lignocellulosic biomass originating from beech wood consists of 1.35% ash, 3.74% extractives, 21.75% lignin, 33.91% hemicellulose and 39.25% cellulose*.

e *ZSM-5 (Si/Al = 100) was commercially purchased*.

f *The result was obtained from NH_3_ temperature programmed desorption*.

g *The ZSM-5 zeolite is synthesized at 160°C for 72 h with raw materials ratio as SiO_2_: Al_2_O_3_: TPABr: Na_2_O: H_2_O = 1: 40: 10: 1.0: 0.02*.

h *ZSM-5 (Si/Al = 46) was commercially purchased*.

i *BETX: sum of benzene, toluene, ethylbenzene, and xylenes*.

j *NM: not measured*.

k *The ZSM-5 is deal with NaOH solution and HCl, with surface area of 354 m^2^/g, total acidity of 103.9 μmol/g*.

#### Catalytic Hydro-Pyrolysis

When hydrogen is added in the pyrolysis process, very active H· radicals will be produced. H· radicals are easy to react to form biomass fragments, while removing oxygen and covering free radicals, thus increasing the hydrocarbons production. Hydro-pyrolysis has been widely used in coal pyrolysis, and the results show that under high hydrogen pressure, cracking, and hydrogenation of bio oil compounds will occur simultaneously (Canel et al., 2005). To study the deoxidization effect of Ni-ZSM-5 catalyst in the process of catalytic hydro-pyrolysis, Thangalazhy-Gopakumar et al. (2012) investigated the catalytic hydro-pyrolysis of pine wood sample (particle size of 149–177 μm) using HZSM-5 and 5 wt.% Ni-HZSM-5 under different hydrogen pressure of 10, 20, 30, and 40 MPa.

**Scheme 1:**
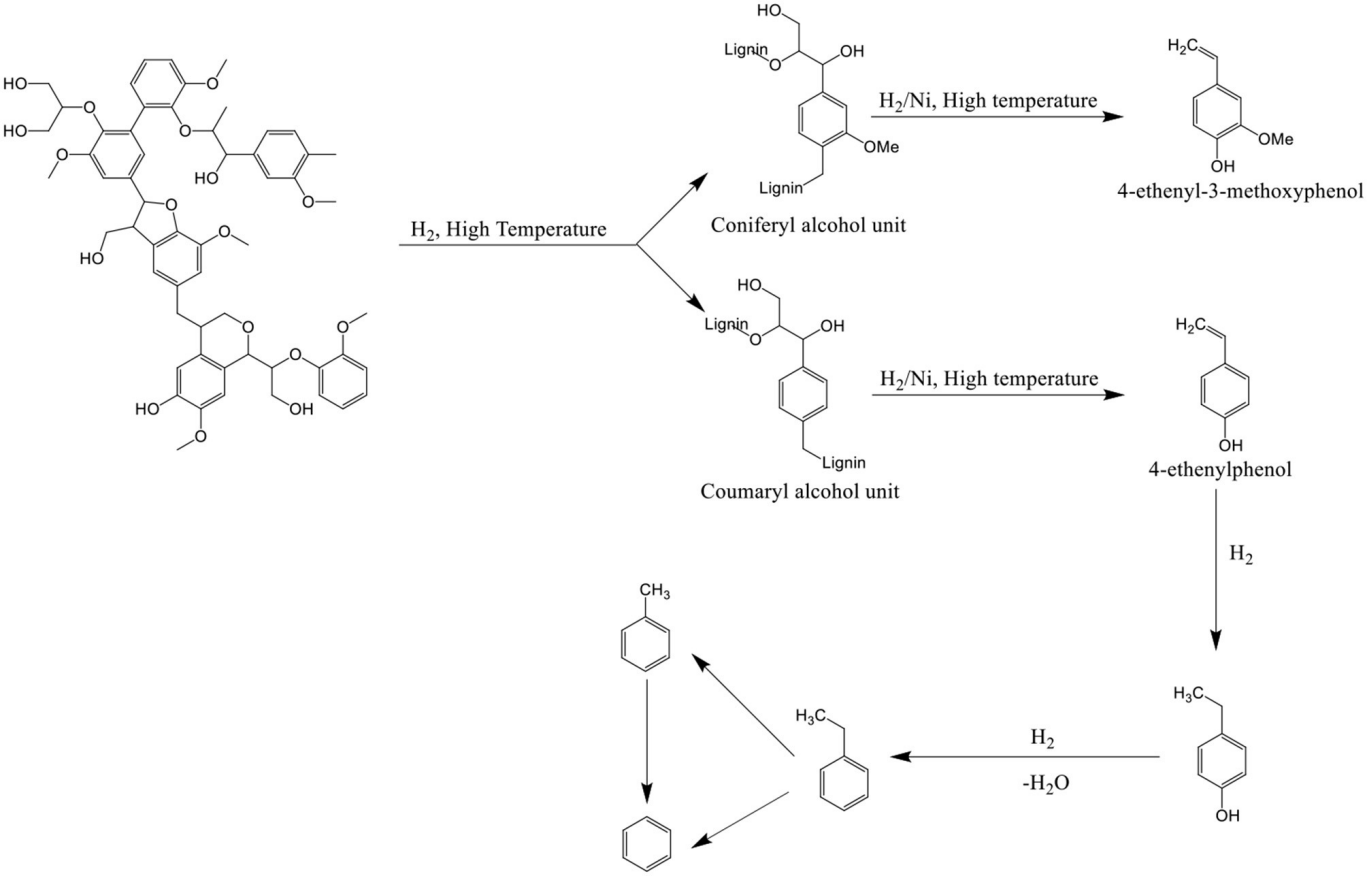
Possible reaction pathway for lignin, from Melligan et al. (2012).

For HZSM-5 catalysts, the aromatic yields have no change in the hydrogen pressure of 10–40 MPa, and the catalyst may only play the role of cracking catalyst, not the role of promoting hydrogenation (Pindoria et al., 1998). Lower amounts of the higher molecular weight phenolic compounds and larger amounts of the lighter phenols were observed in the presence of Ni supported ZSM-5 (Melligan et al., 2012). The possible reaction mechanism for the formation of phenols, benzene, toluene, and ethylbenzene is shown in Scheme 1. Compared with ZSM-5 catalyst, Ni-ZSM-5 lowered the ethanoic acid content, and a further decrease of 20% in ethanoic acid was observed after introduction of H_2_ to the reaction.

The hydrodeoxygenation of guaiacol and 2-phenoxy-1-phenylethanol as model compounds of lignin are performed with different Ni loadings (5, 10, 12.5, 15, 20 wt.%) Ni-ZSM-5 under 5 MPa of hydrogen pressure (Barton et al., 2018). Regardless of Ni loading, the conversions of guaiacol are nearly 100% and three main products are 2-methoxycyclohexanol, cyclohexanol, and cyclohexane. But the time required to reach 100% conversion decreased with increased Ni loading. The Ni-ZSM-5 was also used to investigate the hydrodeoxygenation of vegetable oils to liquid alkane fuels, where the selectivity of *n*-C5-C16 alkane was 71.0% (Na et al., 2012). Porosity and acidity of different Ni-ZSM-5 catalysts and their catalytic hydro-pyrolysis performance are shown in Table 2.

**Table 2:** Porosity and acidity characteristics of catalysts and catalytic hydro-pyrolysis performance of different catalysts.

**Catalysts**	**Surface area (m^2^/g)^a^**	**Acidic properties (μmol/g)**			**Experimental conditions**		**Primary product in liquid phase (wt.%)**	**References**
		**Brönsted acidity**	**Lewis acidity**	**Total acidity**	**Raw material**	**H_**2**_ pressure (MPa)**		
5 wt.% Ni-ZSM-5^b^	292	None	None	None	Pine wood^c^	40	Aromatic compounds (41.3 ± 4.5)	Thangalazhy-Gopakumar et al., 2012
10 wt.% Ni-ZSM-5^d^	331	1,900^e^	None	None	Scots Pine	AP^e^	Low molecule weight Phenols (13.19) Aromatic hydrocarbons (21.86)	Melligan et al., 2012
10 wt.% Ni-ZSM-5^d^	331	1,900^f^	None	None	Mahogany	AP	Low molecule weight Phenols (16.13) Aromatic hydrocarbons (31.67)	Melligan et al., 2012
10 wt.% Ni-ZSM-5^d^	331	1,900^f^	None	None	Miscanthus × giganteus	AP	Low molecule weight Phenols (31.11) Aromatic hydrocarbons (21.73)	Melligan et al., 2012
40 wt.% Ni-ZSM-5^g^	None	None	None	None	Cellulose	2.0	Hexitols (~48.6)	Liang et al., 2012
10 wt.% Ni-ZSM-5	254	None	None	None	Guaiacol	5.0	2-methoxycyclohexanol (12)	Barton et al., 2018
7 wt.% Ni-ZSM-5^h^	173	None	None	None	Methyl hexadecanoate^i^	2.0	*n*-C_5_-C_16_ alkane (71.0)	Na et al., 2012

a *Multi-point BET method*.

b *ZSM-5 is commercially purchased, whose BET surface area is 301.9 m^2^/g*.

c *Pine wood particle size is 149–177 μm*.

d *ZSM-5 (Si/Al = 23) is commercially purchased, whose BET surface area is 425 m^2^/g*.

e *Refers to atmosphere pressure*.

f *The Brönsted acidity was measured by titration method with standardized 0.1 M NaOH (aq) after catalyst equilibrating with 0.1 M NaCl solution*.

g *ZSM-5 (Si/Al = 25)*.

h *The ZSM-5 (Si/Al = 38) was commercially purchased*.

i *The Methyl hexadecanoate was choose as model compound of vegetable oil*.

### Bimetallic Ni Modified ZSM-5

Although the activity of nickel-based catalysts is higher than that of other catalysts, there are still many problems that limit the utilization of Ni-ZSM-5 catalysts, such as carbon deposition. Zhang et al. (2013) observed that 36 wt.% of residue such as coke was deposited on the surface after hydrotreatment. Zhao et al. (2012) observed the deactivation of Ni-ZSM-5 catalysts owing to sintering during the hydrothermal treatment and recycling process as well as partly Ni leaching from the catalyst into water (Zhao et al., 2012). The deactivation of catalysts mainly depends on the zeolite pore structure, acidic properties, reaction temperature, and properties of reactants. The main function of Lewis acid sites is to bind substances to the catalyst surface; Brönsted acid sites is to transfer protons to related compounds to form carbon positive ions, which is considered to be the main reason for coking (Mortensen et al., 2011). The process of carbon deposition is very complex, in which the intramolecular and intermolecular condensation reactions of reactants and/or products play a key role. These condensation reactions usually undergo nearly irreversible second reaction of dehydrogenation or hydrogen transfer to form stable oligomer or graphite carbon, which is difficult to remove from the acid catalyst. Li et al. (2015) investigate the coke deposition on 15 wt.% Ni-ZSM-5 in bio-oil hydrodeoxygenation process under 523–603 K and 2.0 MPa of hydrogen pressure. The results showed that most of the deposited filamentous carbon is converted into carbon-rich flat plate as the temperature rises from 250 to 330°C. The filamentous carbon and carbon-rich flat plate both possess low volatility and solubility and are often retained within pores or on the outer surface of the catalysts, which could potentially block reactant molecules from reaching acid sites and result in catalysts deactivation. The deposition coked at 250°C is soft carbon which is soluble in organic solvents, the deposition coked at 280°C shows that some soluble carbon burned off or is transformed into derivatives with higher molecular weights, the deposition coked at 300°C shows deposited hard coke (or amorphous graphite) which is insoluble in organic solvents, and the deposition coked at 330°C shows deposited graphite or amorphous graphite (Rossetti et al., 2005; Xu et al., 2009). Thermogravimetry analysis of catalysts after hydrodeoxygenation process shows four stages of mass loss from 50 to 250°C, from 250 to 450°C, from 450 to 600°C, and from 600 to 750°C, which corresponds to the loss of moisture, physical absorbents, the formation of soft coke, hard coke, and graphite, respectively.

Bimetallic catalysts often show properties different from those of the corresponding monometallic catalysts (Alonso et al., 2012). Therefore, bimetallic catalysts have also attracted much attention from researchers in recent years. Huynh et al. (2014) discovered that when using monometallic Ni-ZSM-5 and bimetallic Ni-Cu or Ni-Co-ZSM-5 as catalysts, no differences in adsorption capacities were observed irrespective of mono- or bimetallic catalysts with 20 wt.% loading. Thus, the difference of catalytic performance must be related to other reasons besides pore size effect, because all of these samples have similar micropore volumes. After calcination and reduction of Ni-Cu-ZSM-5 and Ni-Co-ZSM-5, Ni-Cu alloy and possible Ni-Co alloy are detected. What should be pointed is that a new phase of NiCo_2_O_2_ is detected in the calcined Ni-Co-ZSM-5. During the H_2_ temperature-programmed reduction (TPR) experiments, the Ni-Cu-ZSM-5 shows lower reduction temperature, possibly due to synergistic interaction between the oxide phases and even physical mixing of NiO and CuO (Carrero et al., 2007; Rogatis et al., 2009), whereas the effect of Co addition to Ni on reducibility is not as strong as that of Cu on Cu-Ni bimetallic precursors. The above TPR results reveal that the addition of Cu and Co promotes the reduction of nickel oxide. For catalytic hydro-pyrolysis of phenol with Ni-Cu-ZSM-5 catalyst, the phenol conversion decreases substantially along with exchange of Ni with Cu, while the selectivity to cyclohexanol increases. Considering the decrease in the number of Ni active sites and acid sites, it can be concluded that the combination of metal and acid sites can effectively remove oxygen from phenol, and tuning of metal sites can provide additional control over the severity and selectivity of hydrogenation. For catalytic hydro-pyrolysis of phenol with Ni-Co-ZSM-5 catalyst, the phenol conversion is complete for 10 wt.% Ni-10 wt.% Co-ZSM-5 and the selectivity toward deoxygenated products (benzene and cyclohexene) is 99.3%, which is probably due to the formation of a NiCo_2_O_4_ spinel phase in the catalyst precursor resulting in the high dispersion of metal species. The Ni-Cu biometallic zeolite also is investigated by Kumal et al., where CuNi/zeolite showed better deoxygenation efficiency than mono-metallic catalysts (Cu/zeolite and Ni/zeolite) and produced comparatively higher percentage of aromatic hydrocarbons at 14.3% and aliphatic hydrocarbons at 39.9% (Kumar et al., 2019, 2020).

The addition of noble metals to Ni-ZSM-5 show excellent catalytic performance in hydrogenation of microcrystalline cellulose (Liang et al., 2014). All investigated noble-metal-modified Ni-ZSM-5 gave enhanced hexitols yields in an order of Ir-Ni-ZSM-5 (49.2%) < Ru-Ni-ZSM-5 (55.6%) < Pd-Ni-ZSM-5 (58.9%) < Rh-Ni-ZSM-5 (60.6%) < Pt-Ni-ZSM-5 (76.9%). The TPR results show that the reduction of NiO is promoted by the addition of noble metals, and this promotion is due to hydrogen spillover, i.e., free hydrogen migrates from noble metals to NiO surfaces. The close interaction between platinum clusters and nickel clusters leads to homogeneous reduction, forming Pt-Ni alloy. In addition, the size of metal particles decreases to 13 nm in the presence of Pt, indicating that the addition of Pt results in higher dispersion of Pt-Ni particles, as same as the effect of addition of Ce (Li et al., 2017). The Pt-enriched alloy surface can inhibit the oxidation of nickel and suppress the leaching of active nickel, which leads to excellent hydrothermal stability. Guo et al. (2019) investigate the cleavage C-O ether bond of lignin model compounds (2-methoxyphenyl anisole, 2-(2-methoxyphenoxy)-1-phenylethanol, and 4-phenoxyphenol) over Ni/CaO-ZSM-5 catalyst and conclude that low H_2_ pressure favored hydrogenolysis, while high H_2_ pressure favored hydrogenation. Porosity and acidity of different bimetallic Ni-ZSM-5 catalysts and their catalytic hydro-pyrolysis performance are shown in Table 3.

**Table 3:** Porosity and acidity characteristics of catalysts and catalytic performance of different bimetallic Ni-ZSM-5.

**Catalysts**	**Surface area (m^2^/g)^a^**	**Acidic properties (μmol/g)**			**Experimental conditions**		**Primary product (wt.%)**	**References**
		**Brönsted acidity**	**Lewis acidity**	**Total acidity**	**Raw material**	**H_**2**_ pressure (MPa)**		
17 Ni 2 Cu^b^	63.0^c^	None	None	None	Phenol	AP^d^	Cyclohexane (77)	Huynh et al., 2014
16 Ni 4 Cu	65.3^c^	None	None	None	Phenol	AP	Cyclohexane (60)	Huynh et al., 2014
10 Ni 10 Co	65.4^c^	162	509	672	Phenol	AP	Cyclohexane (92)	Huynh et al., 2014
5 Ni 14 Co	63.5^c^	166	527	693	Phenol	AP	Cyclohexane (90)	Huynh et al., 2014
17 Ni 1 Pt	None	None	None	None	Cellulose	4.0	Hexitols (76.9)	Liang et al., 2014
1.3 Ni 3.1 Ga	328	None	None	None	Eucalyptus urophylla	None	BTEX^e^ (NM^f^)	Schultz et al., 2016
8 Ni 8 Ce	257	None	None	None	Phenol	None	H_2_ (NM)	Li et al., 2017
45 Ni 5 Ca	None	31.8	157.6	189.4	2-methoxyphenyl anisole	1.0	Phenol (31.3)	Guo et al., 2019
45 Ni 5 Ca	None	31.8	157.6	189.4	2-(2-methoxyphenoxy)-1-phenylethanol	1.0	1-Phenyl ethanol (37.6) Guaiacol (43.7)	Guo et al., 2019
45 Ni 5 Ca	None	31.8	157.6	189.4	4-phenoxyphenol	1.0	Toluene (49.4) Guaiacol (50.5)	Guo et al., 2019

a *Multi-point BET method*.

b *Refers to 17 wt.% Ni 2% Cu-ZSM-5, and the other representations have the same meaning*.

c *Refers to the N_2_ adsorbed volume (cm^3^·g^-1^) at standard temperature and pressure (0.1 MPa, 0°C)*.

d *Refers to atmosphere pressure*.

e *BETX: sum of benzene, toluene, ethylbenzene, and xylenes*.

f *NM, not measured*.

## Outlook

Despite the praiseworthy achievements in catalytic pyrolysis and catalytic hydro-pyrolysis of bio oil have been studied extensively, there are still some disadvantages, such as harsh conditions (high temperature and high pressure), the use of molecular H_2_ as hydrogen donor, and the high cost of developing catalysts (generally noble metal catalyst is needed), low selectivity to target products and low catalyst stability. Special attention should be paid to the stability of bifunctional ZSM-5 catalysts containing different catalytic sites (metal and acid sites), which get deactivated due to different mechanisms. The metal particles on ZSM-5 suffer from leaching out while the ZSM-5 suffers from phase transformation and surface area loss. However, there are limited methods to stabilize each type of catalytic active sites. Though surface functionalization can be used to stabilize ZSM-5 catalyst, it is difficult to prepare bifunctional catalysts by different methods. Considering that improving the stability of catalyst can help to solve the problem of high catalyst cost, further study on the stability of bifunctional sites is needed. It is also necessary to study the long-term operation of continuous reactor system, because the batch reactor used in most studies is not the appropriate choice to evaluate the stability of catalyst. In order to obtain higher quality bio oil, most processes need to add a lot of molecular H_2_, which is usually obtained from non-renewable resources (such as steam reforming of coal). The use of gaseous hydrogen has brought challenges to the process economy and transportation. More importantly, its low solubility in most solvents requires high operating pressure, which will cause serious safety problems.

Excessive metal loading reduces the acidity and physical properties of ZSM-5, and then reduces the diffusivity and catalytic activity of the reactants. High mesopore content will increase coke formation and catalyst deactivation, so it is necessary to determine the mesoporous content of ZSM-5 and the optimal metal loading. Considering the cost of catalyst, the regeneration of ZSM-5 is still the focus of future research. Co-pyrolysis of biomass needs to be taken into account as an effective way to improve the quality as well as quantity of bio-oil, in which two or more materials will be used as feedstock. In general, the use of catalysts for biomass conversion has achieved valuable results, but there is still much room for us to understand the design of catalysts for the selective preparation of target products from biomass. Therefore, the reasonable design of catalyst is of great significance to the innovation of biomass conversion technology.

## Author Contributions

R-QL: conceptualization and supervision. Y-LD and H-QW: investigation and original draft. Q-PK: methodology. MX and PY: review and editing. All authors: contributed to the article and approved the submitted version.

## Conflict of Interest

The authors declare that the research was conducted in the absence of any commercial or financial relationships that could be construed as a potential conflict of interest.
